# Development of antimicrobial peptides for catheter-related bloodstream infectionprevention

**DOI:** 10.1186/2047-2994-4-S1-I4

**Published:** 2015-06-16

**Authors:** TA Camesano, T Alexander, L Lozeau

**Affiliations:** 1 Chemical Engineering, Worcester Polytechnic Institute, Worcester, USA

## Introduction

Central venous catheters are used in many medical procedures to deliver critical and lifesaving treatments, such as antibiotics and chemotherapy agents. However, central line-associated bloodstream infections (CLABSIs) associated with these devices negatively impact over 100,000 patients per year and are extremely expensive to treat.

When infections arise on implanted biomaterials, they are treated with systemic antibiotics, debridement, and implant removal. Unfortunately, the high local antibiotic concentrations needed to kill colonized bacteria are only achieved over a short time, cause cytotoxicity, and can promote antibacterial resistance. Therefore, it is currently believed that proactive methods of infection management will be superior to reactive methods.

As an alternative to traditional antibiotics or to device removal, antimicrobial peptides (AMPs) represent a novel way to prevent and treat infections. AMPs are short, cationic molecules found naturally in the innate immune systems of many species, and they have broad spectrum antimicrobial activity. AMPs use fundamentally different mechanisms to kill bacteria than conventional antibiotics, reducing the threat of bacterial resistance.

## Objectives

The objective of our overall research program is to improve biomedical devices using a therapy based on surface-tethered AMPs. Although AMPs are highly active against bacteria when free in solution, immobilization of AMPs to a surface significantly reduces antimicrobial activity.

## Methods

Antimicrobial peptides are tethered to biomaterial surfaces using flexible chemical linkers. We are currently investigating the role of the linker size in influencing the efficacy of the antimicrobial peptide. Activity against Gram-negative and Gram-positive bacteria is being quantified. We are also studying the cytotoxicty and long-term stability of the tethered peptides.

## Results

We have shown that modified antimicrobial peptide, chrysophsin-1, can be chemically tethered to a biomaterial surface using polyethylene glycol as a spacer. Killing was observed for *Staphylococcus aureus* and *Escherichia coli*.

## Conclusion

The expected outcome of this project is a surface modification on catheters can be used to help mitigate catheter-related bloodstream infections.

## Disclosure of interest

None declared.

